# Survival in myotonic dystrophy type 1: a long time follow up-study with special reference to gastrointestinal symptoms

**DOI:** 10.48101/ujms.v129.10663

**Published:** 2024-09-17

**Authors:** Anders Rönnblom, Anders Ekbom

**Affiliations:** a Department of Medical Sciences, Uppsala University, Uppsala, Sweden; b Clinical Epidemiology Division, Department of Medicine Solna, Karolinska Institutet, Stockholm, Sweden

**Keywords:** DM1, gastrointestinal symptoms, survival

## Abstract

**Background:**

Myotonic dystrophy type 1 (DM1) is a monogenetic disease affecting many organs. Gastrointestinal symptoms are prevalent and of considerable consequences for affected individuals. The life expectancy is shortened and the objective of the study is to evaluate if gastrointestinal symptoms can predict the outcome of the disease.

**Method:**

Fifty-one patients with DM1 were interviewed regarding symptoms from the gastrointestinal tract in the mid-1990s. Survival of all patients was evaluated in 2023 and the impact of symptoms on survival was assessed.

**Results:**

At the beginning of the study, the mean age was 35.9 years, (median 37.0, 9–63). At the end of the study 47 out of the 51 patients were deceased at a mean age of 53.7 years (median 55.7, 32.5–79.0). Patients with the congenital form of DM1 (*n* = 6) died at an age of 46.0 years (median 45.2, 40.0–53.6). There was no correlation between the gastrointestinal symptoms and survival.

**Conclusion:**

Albeit prevalent and of considerable clinical consequence, gastrointestinal symptoms are not correlated to survival in myotonic dystrophy type 1.

## Introduction

Myotonic dystrophy type 1 (DM1) is an autosomal dominantly inherited disease with highly variable penetrance. The prevalence is estimated to be 12 in 100,000 individuals (1), but in Norrbotten, the northernmost county of Sweden, the prevalence was calculated to be at least 70/100,000 (2). This disease was described as a separate disease entity during the last years of the 19th century and it was called myotonia atrophica (3). Later the name myotonic dystrophy was applied to the disease, but the discovery of similar diseases with other genetical aberrations made it necessary to further develop the terminology (4). The patients described in this report were diagnosed as myotonic dystrophy at the inclusion of the study but are now classified as DM1.

The life expectancy in DM1 has been reported to be shortened (5–9). More than 50% of deaths are related to progressive neuromuscular disease resulting in respiratory failure. Cardiovascular disease is the second most common cause of death (5, 8). Since gastrointestinal symptoms are common among patients with DM1, i.e. swallowing difficulties and coughing while eating, nausea, abdominal pain and anal incontinence (10), it could be speculated that patients suffering from these symptom could be at an increased risk of a premature death due to both aspiration or impaired nutrition.

This study is based on a cohort of patients with DM1 from northern Sweden who were studied in the mid-1990s with special reference to gastrointestinal symptoms (10).

The aim of the study is to evaluate if a careful clinical interview of gastrointestinal symptoms could predict a shortened survival in patients suffering from DM1.

## Patients and methods

### Patients

Norrbotten is the northernmost county of Sweden, with 265,000 inhabitants when the study started. Early after the description of the disease, it was reported that it could be found in Norrbotten County (11). Fifty-one patients underwent a broad interdisciplinary team assessment and were interviewed specifically with respect to gastrointestinal symptoms and classified according to their functional capacity. Six individuals suffered from congenital DM1. The diagnosis was based on clinical grounds and all had a positive family history. The patients represent different severities of disease: *mild*, with symptoms but no functional disturbances in activities of daily life; *moderate*, with some functional disturbances that, however, do not prevent the performance of normal daily activities and permit the patient to manage a light job; and *severe*, with major functional disturbances that prevent the patient from carrying out most normal daily activities (2). The study started a few years after the genetic background for the disease was described and only a few of the individuals were investigated with respect to this finding (12).

### Interdisciplinary team assessment

An interdisciplinary team was organised at the hospital in Boden (13). All patients with DM1 residing in Norrbotten County were invited to be assessed by the team. The team comprised a neurologist, a cardiologist, a gastroenterologist, a nurse (also team coordinator), an occupational therapist, a physical therapist, and a social worker. The aim of the interdisciplinary team assessment was to determine the patients´ functional capacity and identify any medical problems. The actual assessment took place during 2 days, where the patient met individually with each team member. The team then met together with the patient and summarised the assessment and formed a plan for the different interventions. The intention with the different interventions, tailored to each individual, was to reduce their self- perceived disability and plan for the future. Each patient was then offered individual follow-up according to the plan.

### Interview of gastrointestinal symptoms

The patients’ sex- and age-matched controls were interviewed with a questionnaire covering symptoms from all parts of the gastrointestinal tract. Respondents were asked whether symptoms were lacking, occurred occasionally but not more than once a week, more than once a week, or daily. A control group of 40 subjects consisting mainly of healthy hospital workers were interviewed with the same questionnaire. The interviews were performed between 1992 and 1995. Information regarding survival was obtained via the Swedish Total Population Register (TPR) in 2023.

### Statistics

All data analysis were performed using the software STATISTICA (version 10; 2011; StatSoft Inc., Tulsa, OK; http://www.statsoft.com). Non-parametric continuous variables are presented as means and medians and were tested for significance with the Mann–Whitney test whereas categorical variables were tested with the χ^2^-test. *P* < 0.05 was considered significant. Survival was tested for by the Log-Rank test, after stratifying for early onset and later, and the different gastrointestinal symptoms.

### Ethical considerations

The study was approved by the local Ethics Committee at Uppsala University (diary number 2023-01411-01).

## Results

The clinical description of patients and controls are presented in Tables 1 and 2. Most prevalent symptoms in the patients in comparison with the controls were dysphagia, coughing while eating, vomiting, and abdominal pain (Table 3). Regarding bowel habits, diarrhoea was particularly common and more than half of the patients reported at least one episode of pneumonia earlier in life. (Table 4).

**Table 1:** Clinical characteristics of patients with myotonic dystrophy type 1

Gender	No	Clinical form	No	Severity	No
Male	26	Congenital	6	Mild	1
Female	25	Classical	45	Moderate	24
				Severe	10
				Missing	2
Total	51		51		51

**Table 2:** Body measurements and age at inclusion

	Patients (*n* = 51)	Controls (*n* = 40)	*P*-value*
Length (m, Mn ± sd)	1.66 ± 0.09	1.73 ± 0.11	0.002
Weight (kg, Mn ± sd)	64.1 ± 15.3	71.9 ± 15,3	0.01
BMI (Mn ± sd)	23.1 ± 5.0	23.8 ± 4.0	0.3
Age (Md, yrs), all	37.0 (9–63)	36.0 (11–53)	0.9
Age congenital MD1	21.5		

* Mann–Whitney test.

**Table 3:** Gastrointestinal symptoms in 51 patients with myotonic dystrophy

	Symptom present			Symptom present more than once a week		
	Patients (51)	Controls (40)	*P*	Patients (51)	Controls (40)	*P*-value*
Dysphagia	23	6	0.002	12	0	0.001
Coughing while eating	19	7	0.038	9	0	0.005
Choking	5	1	0.16	2	0	0.21
Need to clear throat	6	0	0.025	5	0	0.041
Heartburn	22	15	0.59	5	1	0.16
Regurgitation	10	8	0.92	3	0	0.12
Nausea	18	4	0.005	9	1	0.022
Vomiting	12	0	0.001	8	0	0.009
Early satiety	10	1	0.013	9	0	0.005
Abdominal pain	30	7	0.001	15	3	0.009
Anal incontinence	13	0	0.001	6	0	0.025

* χ^2^-test.

**Table 4:** Bowel habits, pneumonia and urinary incontinence

	Patients (*n* = 51)	Controls (*n* = 40)	*P*-value*
Constipation	7	0	0.02
Diarrhoea	16	3	0.005
Pneumonia	27	6	0.0002
Urinary incontinence	8	2	0.1

* χ^2^-test.

**Figure 1:**
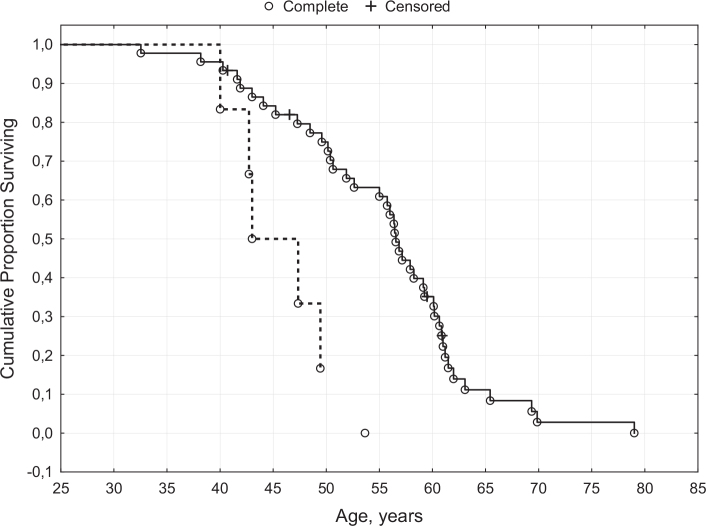
Survival of patients with myotonic dystrophy type 1. Solid line = surviving patients with adult DM1 Dotted line = surviving patients with congenital DM1

Only four patients were still alive at this follow-up. The median age at death was 55.7 years (range 32.5–79.0). Patients with the congenital form of the disease died at a lower age (45.2 years vs. 56.4, *P* = 0.01), Figure 1. Survival evaluated with the Log-Rank test demonstrated a significant difference in survival, *P* = 0.04. None of the evaluated gastrointestinal symptoms was associated with a reduced survival. Neither could a history of a pneumonia predict survival (data not shown).

## Discussion

The main finding in this study is the prevalent occurrence of gastrointestinal symptoms among patients with DM1, the reduced life expectancy but the absence of any correlation between these observations. The congenital form of the disease has a shortened life expectancy compared to patients with a later disease manifestation.

At the inclusion of the study, the median age of the patients was 36.5 years and the median age at death 55.7 years. This could be compared with the average Swedish population, which demonstrates a residual life expectancy of approximately 50 years for individuals of 30 years of age (14).

In a 10-year study in a cohort of patients with DM1, 20% of the patients died during the observation time, and the predominant reason was respiratory problems (7). The mean age at death was 53.2 years, range 24–81. None of the patients with congenital DM1 died during the observation. The congenital form of the disease was studied in 115 patients born between 1940 and 1989 (9). In this study, 25% of the patients died within 18 months, mainly in the neonatal period, as a result of respiratory complications. Half of the individuals survived to their mid-thirties but then died before reaching 41. A later study where all patients were analysed genetically, demonstrated an inverse relationship between survival and the length of the CTG repeat (5). The median survival in this study was 55 years and more than 50% of the deaths were related to progressive neuromuscular disease resulting in respiratory failure. Similar findings were reported from an Italian single centre study with worse outcome in patients with longer CTG expansion (8). The mortality in the study was however low (12.1%), probably because of a short observation time.

A scoring system to predict survival in DM1 has been developed (15). A derivation cohort of 1,066 patients with a mean age of 39.3 years was studied and extracted variables were evaluated in a validation cohort of 230 patients with a mean age of 41.9 years. The most important variable was age above 45 years HR 6.82 (4.20–11.1), but additional information was added if the vital capacity was ≤60% of predicted value with a of HR 2.32 (1.63–3.30) and a need for walking support, HR 1.88 (1.27–2.79). Other factors had less impact on survival. Dysphagia was included in the scoring system but had no effect on the score.

Although gastrointestinal symptoms are common and of considerable importance for the well-being of these patients (10), we could not demonstrate any impact on survival for any of the studied symptoms. A possible explanation could be that respiratory failure evolves independent of gastrointestinal symptoms and that a lethal cardiac arrhythmia can affect an otherwise rather healthy individual.

## Strengths and limitations

The strength of this study is the careful description of gastrointestinal symptoms in a cohort of patients suffering from DM1, which has been followed for more than 25 years, where all except four have reached the targeted endpoint. The use of the Swedish Total Population Register as a method of evaluation leads to a 100% coverage of survival.

The study is limited by an absence of genetical information and causes of death for the individuals studied. The relatively small study group results in a potential for a type II error. The control group is probably healthier than the general population.

## Conclusion

DM1 is a systemic disease with many organ manifestations, among whom gastrointestinal symptoms are common. The life expectancy is shortened, but gastrointestinal symptoms do not predict survival.

## Data Availability

The data that support the findings of this study are available on request from the corresponding author. The data are not publicly available due to privacy or ethical restrictions.
